# Experimental Study: Deep Learning-Based Fall Monitoring among Older Adults with Skin-Wearable Electronics

**DOI:** 10.3390/s23083983

**Published:** 2023-04-14

**Authors:** Yongkuk Lee, Suresh Pokharel, Asra Al Muslim, Dukka B. KC, Kyoung Hag Lee, Woon-Hong Yeo

**Affiliations:** 1 Department of Biomedical Engineering, Wichita State University, Wichita, KS 67260, USA; amalmuslim@shockers.wichita.edu; 2 Department of Computer Science, Michigan Technological University, Houghton, MI 49931, USA; sureshp@mtu.edu (S.P.);; 3 School of Social Work, Wichita State University, Wichita, KS 67260, USA; kyoung.lee@wichita.edu; 4 George W. Woodruff School of Mechanical Engineering, Georgia Institute of Technology, Atlanta, GA 30332, USA; whyeo@gatech.edu; 5 IEN Center for Human-Centric Interfaces and Engineering, Georgia Institute of Technology, Atlanta, GA 30332, USA

**Keywords:** skin-wearable electronics, fall monitoring, deep learning, older adults

## Abstract

Older adults are more vulnerable to falling due to normal changes due to aging, and their falls are a serious medical risk with high healthcare and societal costs. However, there is a lack of automatic fall detection systems for older adults. This paper reports (1) a wireless, flexible, skin-wearable electronic device for both accurate motion sensing and user comfort, and (2) a deep learning-based classification algorithm for reliable fall detection of older adults. The cost-effective skin-wearable motion monitoring device is designed and fabricated using thin copper films. It includes a six-axis motion sensor and is directly laminated on the skin without adhesives for the collection of accurate motion data. To study accurate fall detection using the proposed device, different deep learning models, body locations for the device placement, and input datasets are investigated using motion data based on various human activities. Our results indicate the optimal location to place the device is the chest, achieving accuracy of more than 98% for falls with motion data from older adults. Moreover, our results suggest a large motion dataset directly collected from older adults is essential to improve the accuracy of fall detection for the older adult population.

## 1. Introduction

Falls create a serious public health issue among older adults aged 65 years or above. Aging limits the ability to move the body effectively, so falls can cause significant injuries and even mortality, and can consequently result in enormous costs for healthcare services [1,2,3]. A study showed that falls are the direct or indirect cause of approx. 62% of injury-related hospitalizations for older adults in Canada [4]. Another study demonstrated that over 12,000 older adults died, and approx. 1.7 million older adults were treated in the emergency room as a result of falls in 2002 in the United States [5]. In general, approx. 28~35% of older adults over 65 years old and 32~42% of older adults over 70 years old experience fall-related injuries more than one time each year [6]. The number of older adults suffering from fall-related injuries will gradually increase as time goes on since their population is growing faster than any other age groups. Their population was 49.2 million in 2016 (approx. 15% of the U.S. population) and is expected to reach 98 million by 2060 (approx. 25% of U.S. population) [7]. The costs of falls and fall-related injuries have already impacted the U.S. healthcare system. Approx. 6% of Medicare expenses, 8% of Medicaid expenses, and 5% of other sources of payment including private insurance were spent on fall-related injuries for older adults in 2015 [8], and their medical costs are estimated as USD 56 billion by 2020 [9]. To minimize the adverse consequences of falls and provide adequate medical responses and cares for older adults, a cost-effective, reliable, and immediate fall detection system is essential.

The first fall detection system developed in the 1970s was designed to send an alarm when a user pressed a remote transmitter button [10]. Currently, there are ongoing efforts toward the development of automatic fall detection systems [11,12,13]. Automatic fall detection systems can be divided into two categories: wearable and non-wearable systems. Non-wearable systems utilizing cameras [14,15,16,17], microphone arrays [18,19], floor pressure sensors [20,21,22], or floor vibration sensors [23,24,25] can provide sufficient information on human movements, along with a high percentage of sensitivity and specificity. For example, Patsadu et al. achieved the accuracy of 99.97% in fall detection with zero false negatives using Kinect’s 3D depth camera [26]. Ali et al. achieved the accuracy of 99.2% in fall detection with the Multiple Cameras Fall dataset [27]. However, the major disadvantages of those systems are the complex setup, high cost, and area constraints. In contrast, most wearable-based fall detection systems using accelerometers and/or gyroscopes are relatively cost-effective and easy to use in both indoor and outdoor settings, especially for older adults who are fully independent [11]. For example, He et al. claimed 95.67% accuracy in fall detection using Bayes network classifier, where acceleration and gyroscope data were collected from people aged 20–45 years wearing a vest with the sensor board placed [28]. Saleh et al. reported up to 99.94% accuracy with support vector machine-based fall detection, where acceleration data were acquired by a motion sensor mounted on the waist [29]. Overall, traditional wearable systems seem promising as cost-effective, reliable, and immediate fall detection systems, but there are some limitations. Traditional wearable systems are housed in a rigid plastic board and carried on a belt, band, or in the form of a necklace [30,31,32], which often cause significant visual/body discomfort and generate undesired signals preventing long-term use and/or accurate measurements [33,34]. As a result, there is a dramatic loss in their performance in real-world scenarios, even though their performance is impressive in laboratory environments [35]. Their common placements are mainly the upper body, including the head, neck, chest, trunk, waist, and wrist [32,36,37,38,39], but studies show conflicting results on the optimal placement of wearable devices on the human body for fall detection. For instance, Kangas et al. demonstrated that the waist and head are efficient positions [40], while Bagnasco et al. reported that the chest is the optimal placement of wearable devices for fall detection [41]. Finally, many studies have utilized motion data collected from younger age groups to develop their fall detection algorithms, and there are limited studies with motion data collected from older adults.

To address the aforementioned limitations and develop a comfortable and reliable fall detection system, here, we performed experimental studies, including the development of a wireless skin-wearable motion monitoring (SWM) device for fall detection. The newly developed device exhibited low-profile, ultrathin, and flexible construction, allowing it to form an intimate integration on the skin via van der Waals interactions. As a result, it showed maximum compliance to natural skin motions without causing much discomfort and was easily placed onto the skin of different body locations, such as the wrist and chest. Besides its skin-like mechanical properties, the combination of the flexible circuit with electronic chips delivered key features, including precise motion tracking using a six-axis inertial measurement unit (IMU) and wireless data transmission via Bluetooth technology. The device was wirelessly connected to a mobile Android device, such as a smartphone or tablet PC, to collect the raw motion data for post-analysis. Furthermore, the flexible device was fabricated with commercially available ultrathin copper films, so the manufacturing process and costs were dramatically reduced.

In addition to the development of the skin-wearable device, we attempted to develop a deep learning-based fall detection method for older adults using the notion of a multiclass imbalanced classification problem, where the training dataset consisted of five different human activity classes (e.g., walk, stair, run, sit, and fall) and the distribution of examples across these classes was not equal. While machine learning has been widely used to improve the sensitivity and specificity in fall detection [42,43,44,45], deep learning-based approaches can be more effective to solve time series classification problems, such as human activity recognition [46,47,48,49,50]. Deep learning models are capable of deriving relevant features from raw data without domain knowledge. In addition, they use neural networks with multiple layers to learn complex patterns, often leading to more accurate results [51,52]. We explored the combination of various input datasets from different body placements of the skin-wearable device and trained several commonly used deep learning models for effective fall detection. Those results indicated that (1) the LSTM model that uses XYZ acceleration and gyroscope input dataset achieves the highest accuracy for fall detection (e.g., approx. 97.6% for young adults and 98.5% for older adults), and (2) the optimal location to place the device for fall detection is the chest with an accuracy of 97.6%, followed by the necklace with an accuracy of 94.8% and the wrist with an accuracy of 94.4%. Additionally, our cross-testing results on data collected from different age groups suggested that a large motion dataset collected directly from older adults is essential to improve the accuracy of fall detection for the population of older adults. Overall, our findings will contribute to the development of a cost-effective, reliable, and immediate fall detection system in real-world scenarios and the improvement of the life quality of older adults.

## 2. Materials and Methods

**Figure 1:**
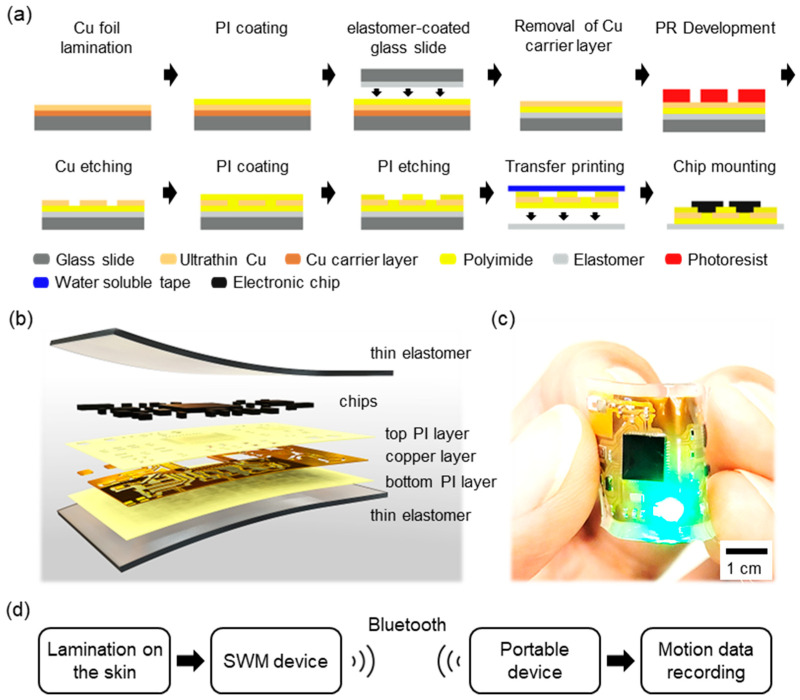
The overview of the skin-wearable device. (**a**) Illustration of device fabrication process using an ultrathin Cu film. (**b**) Exploded view of the device showing materials for each layer. (**c**) Device bending. (**d**) The flow of motion data from the skin to analysis.

**Device fabrication:** Figure 1 includes the overview of device fabrication. The fabrication of a flexible circuit for the SWM device started with the fixation of a 3.5″ × 2.5″ copper (Cu) foil (2 μm-thick MicroThin^TM^ MT18FL; Oak Mitsui Technologies LLC, Frankfort, KY, USA) on a 7″ × 5″ glass substrate. The Cu foil is composed of two Cu layers: a 2 µm-thick ultrathin Cu layer and an 18 µm-thick carrier Cu layer. The Cu foil was fixed in the way that the ultrathin Cu layer was faced up. Approx. 18 µm-thick polyimide (PI; HD MicroSystems LLC, Parlin, NJ, USA) as a supporting layer was spin-coated on the ultrathin Cu layer. Once the PI layer was fully cured, a 3″ × 2″ glass slide coated with a thin elastomer (SORTA-ClearTM 12; Smooth-on, Inc., Macungie, PA, USA) was placed on the top of the PI layer. The thin elastomer was used to facilitate the release of the fabricated circuit during the materials transfer printing process due to the non-interaction between the elastomer and the fully cured PI layer. The Cu foil was cut along the glass slide after the elastomer was cured, and the careful removal of the carrier Cu layer was followed to develop the circuit pattern with photolithography (AZ4620; Microchemicals GmbH, Ulm, Germany) and Cu wet etching (APS-100; Transene Company, Inc., Danvers, MA, USA) on the ultrathin Cu layer. Once the etching was completed, a 2.0 μm-thick PI layer was spin-coated on the top of the circuit as an insulation layer. Openings, where electronic components were mounted, were created along the circuit pattern using a reactive ion etcher (RIE at 150 W and 400 mTorr of O_2_ gas; Jupiter III; March Instruments, Inc., Anaheim, CA, USA). The assembly process of the device included the following steps: (1) the fabricated flexible circuit from the elastomer-coated glass slide was retrieved and transferred onto a thin elastomeric membrane; (2) surface mount electronic components were mounted on the flexible circuit using a low-temperature solder paste (SMDLTLEP, Chip Quik, Niagara Falls, NY, USA); and (3) device functionalities were confirmed before encapsulation with a thin coating of elastomer.

**Motion data collection:** Motion data were collected from four different groups using the SWM device. Each group included 5 participants. One group consisted of people aged 65 and above, and the other three groups consisted of people aged 21–30. To test an optimal device location, each young adult group wore the SWM device on the upper chest or wrist or as a necklace during motion data collection. To compare motion data between different age groups, motion data were collected from the older adult group, while they wore the device on the upper chest. Each participant performed four different daily human activities including standing up/sitting down, walking, running, climbing stairs up/down, and three different types of falls such as forward, backward, and lateral falls. All falls were performed on a large airbed, and instructions and demonstration for each type of falls were provided to participants before fall data collection to prevent any injuries. The total motion data points from all activities were 2894, 3104, 4326, and 3559 for the upper chest, wrist, and necklace of the young adult groups and the upper chest of the older adult group, respectively, presented in Table 1. It can be observed that the dataset is imbalanced and hence is a multiclass imbalanced classification problem. Furthermore, since we are handling each frame as a sequential time series data, we converted the overall data into frames of 2 s as preprocessing steps to train the model. For each activity and each group, available data were divided into a train and test set in the ratio of 80% and 20% of them, respectively. After the motion data collection, the older adult group participated in a survey to inquire about device use perception. All data collection with human subjects was conducted at Wichita State University, following the institutional review board-approved protocol (WSU IRB approval number: 4759).

**Deep Learning models:** For falls and other human activity detections, five various neural network architectures: Long Short-Term Memory (LSTM), 1-Dimensional Convolutional Neural Network (CNN-1D), 1-Dimensional Convolutional LSTM (ConvLSTM-1D), Bidirectional LSTM (Bi-LSTM), and CNN-LSTM, were explored in this study. Those model architectures were trained and tested with four different combinations of inputs: (1) magnitude of acceleration (acc. Mag., 1 attribute); (2) XYZ acceleration (XYZ acc., 3 attributes); (3) XYZ acceleration and gyroscope (XYZ acc. and gyro., 6 attributes); and (4) XYZ acceleration, gyroscope, and magnitude of acceleration (combined, 7 attributes), which collected the different body locations described above. The magnitude of acceleration ($a_{m}$) is calculated by:

(1) $$a_{m}=\sqrt{a_{x}{}^{2}+a_{y}{}^{2}+a_{z}{}^{2}}$$

where $a_{x}$, $a_{y}$, and $a_{z}$ are acceleration values in directions of the *x*-, *y*-, and *z*-axis, respectively. Each model architecture was optimized using cross-validations and grid search over a wide range of hyperparameters. All the hyperparameters regarding model selection and optimization are presented in Table S1. Since the number of data points per class is not distributed equally, the predictive capability of the model trained on such an imbalanced dataset may exhibit poor predictive performance, specifically for the minority classes. To address this problem, we utilized one of the cost-sensitive learning algorithms called the class weighting method which penalizes the different costs for the misclassifications in the majority and minority classes while training the model. The weight for the *j*th class is calculated as *w_j_ = N/(C ȗ n_j_)*, where *N* is the total number of data points, *C* is the number of classes, and *n_j_* is the number of data points in class *j*. The class weight calculated for young adult chest data for various five classes is presented in Table S2.

## 3. Results and Discussion

**System Overview:** Figure 1a briefly illustrates our device fabrication process including ultrathin Cu film preparation, microfabrication, material transfer printing, and chip mounting. Actual images of the device fabrication process can be found in Figure S1. The fabricated SWM device for fall detection included a multilayered flexible circuit and rigid chip components, encapsulated together using a thin silicone membrane. The size of the flexible circuit was 26 mm × 18 mm, and it was composed of two dielectric PI layers and one conductive Cu layer as shown in Figure 1b. Commercialized ultrathin Cu films were utilized to fabricate the flexible circuit instead of thin film deposition techniques, which made the device fabrication process cost-effective and time efficient. The overall thickness of the circuit was maintained at less than 25 µm before encapsulation in order to demonstrate a high level of flexibility and conform to the curvilinear surfaces of the skin (Figure 1c). The device contained an RF antenna, Bluetooth Low Energy module (nRF52832, Nordic Semiconductor), and a nine-axis inertial measurement unit (MPU-9250, InvenSense Inc., San Jose, CA, USA) operating at 33 Hz for continuous wireless transmission of detected motion data (see Figure S2 for more details). A home-fabricated Android application displayed the motion data in real-time and internally stored those data for any post-analysis (Figure 1d). A small rechargeable lithium polymer battery (30 mAh; 14 mm × 10 mm) was used to power the circuit, and the battery test indicated it lasts up to 3.5 h in continuous operation. Based on the battery test, the power consumption of the device was estimated 28.3 mWh at 3.3 V, which is reasonable when considering the power consumption of nRF52832 (5.3 mA) and MPU9250 (3.2 mA) at their normal mode. The thickness of the elastomeric membrane encapsulating the circuit was carefully optimized since it has great influence on device handling and conformality of the device on the skin. For example, the device with thinner elastomeric membranes shows higher adhesion energy to the skin, while it is extremely difficult to handle without causing damage [53]. In this study, the thickness of encapsulated devices was maintained at approx. 500 µm to provide an adequate level of device handling. The physical and mechanical properties of the membrane were tuned by mixing two different types of silicone elastomers, Ecoflex^TM^ 00-30 and Ecoflex^TM^ GEL (Smooth-On, Inc., Macungie, PA, USA). As a result, Young’s modulus of the membrane was as low as approx. 10 kPa [54], and the tackiness inherited from Ecoflex^TM^ GEL with an ultrathin and lightweight configuration of the device facilitated a tight integration of the device on the skin without any adhesive.

**Figure 2:**
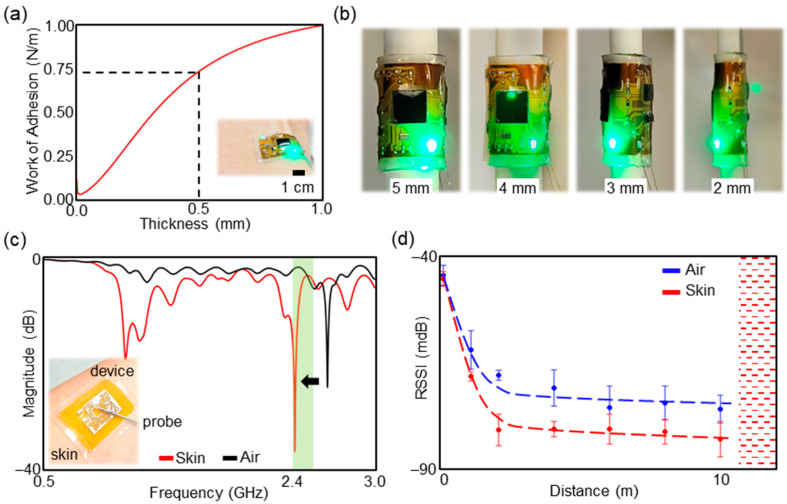
Mechanical and electrical characteristics of the SWM device. (**a**) Relationship between work of adhesion of the elastomeric membrane and the thickness of the encapsulated SWM device allowing the device to form conformal contact with the skin. The thickness of flexible circuit is negligible compared with the thickness of the encapsulated elastomeric membrane. (**b**) Demonstration of device bending on 3D printed rods with different diameters (e.g., 4, 6, 8, and 10 mm). (**c**) Reflection coefficients of the SWM device when it was on the skin and air. (**d**) RSSI measurements of Bluetooth signals transmitted from the SWM device when it was on the skin and air. It demonstrated the device offers the acceptable Bluetooth signal range to monitor motion data from the SWM device using a portable device.

**Device Characteristics:** The SWM device is designed in a way to achieve a high level of mechanical compliance such that the device can provide conformal contact with the skin but also the continued successful operation of the device during user’s activities. An analytical model of the interfacial mechanics indicates conformal contact on the skin occurs when the adhesion energy of the device is larger than the sum of the elastic energy of the skin and the bending energy of the device [53,55]. In our case, the adhesion energy is proportional to the work of the adhesion of the elastomeric membrane ($\gamma_{elastomer}$) encapsulating the device. The skin’s elastic energy depends on the Young’s modulus of skin ($E_{skin}$), skin’s roughness amplitude ($h_{rough}$), and wavelength ($\lambda_{rough}$) when we assume the skin surface as a sinusoidal form. In addition, the bending energy of the device is proportional to an effective bending stiffness (EI) [55]. According to Wang’s work, the simplified expression of the interfacial mechanics with $\gamma_{elastomer}$ can be described as [55]:

(2) $$\gamma_{elastomer}>\frac{\pi^{4}\cdot E_{skin}\cdot EI\cdot h_{rough}{}^{2}}{E_{skin}\cdot \lambda_{rough}{}^{3}+16\pi^{3}\cdot EI\cdot \lambda_{rough}}$$

and

(3) $$EI=\frac{\left(\alpha \cdot E_{circuit}+\left(1-\alpha \right)\cdot E_{elastomer}\right)\cdot {\left(h_{circuit}+h_{elastomer}\right)}^{3}}{12}$$

where α is the fraction of cross-sectional area of the flexible circuit and elastomeric membrane. Since the Young’s modulus of the flexible circuit ($E_{circuit}$) is much higher than the Young’s modulus of the elastomeric membrane ($E_{elastomer}$), we estimated the effective Young’s modulus of the device as $\alpha \cdot E_{circuit}+\left(1-\alpha \right)\cdot E_{elastomer}$ [56]. When we assume skin conditions as $E_{skin}$ = 130 kPa, $h_{rough}$ = 90 µm, and $\lambda_{rough}$ = 180 µm [57,58] and estimate $E_{circuit}\approx E_{PI}$ = 2.5 GPa, $E_{elastomer}$ = 10 kPa, and $h_{circuit}$ = 25 µm, the Equation (2) provides a relationship between desired work of adhesion of the elastomeric membrane ($\gamma_{elastomer}$) and the thickness of the elastomeric membrane ($h_{elstomer}$) that allows the device to form conformal contact with the skin as shown In Figure 2a. In the previous report, the work of the adhesion of the elastomeric membrane was measured as approx. 0.75 N/m [54], so the thickness of the elastomeric membrane ($h_{elastomer}$) for our device was chosen as 500 µm. As a result, not only was the device easy to handle and apply onto the skin, but it also provided the intimate integration on the skin without additional tapes and adhesives.

While the device was naturally adhered to the skin, the flexibility of the device allowed the device to operate seamlessly during normal human activities. To characterize the flexibility of the device, the SWM device was placed on 3D printed rods with different diameters (e.g., 4, 6, 8, and 10 mm) allowing 180° mechanical bending, while the electric power was supplied. The bending radius of the curvature of the device varied upon the region of the device due to the presence of rigid ICs, but the tests demonstrated that the minimum bending radius and overall bending radius of the device are approx. 2 mm and 4 mm, respectively, as shown in Figure 2b. Another bending tests where the SWM device was placed on the middle of a hinge fabricated by two 1 mm-thick glass slides demonstrated the minimum bending radius of the device is approx. 1 mm without device failure (see Figure S3 for more details). As a result, the device is flexible enough to wrap around any adult finger without a significant amount of mechanical stress. In addition to mechanical compliance of the device, the bending tests also showed electrical stability of the device. The microcontroller of the device was programmed in a way that the green LED is on when the device is functional (e.g., receiving motion data from the inertial sensor and transmitting the data wirelessly). During the bending tests, the status of the green LED was carefully observed, and the device was connected to a Tablet PC for wireless data transmission. Those observations indicated that there is negligible effect in terms of device’s functions with given bending strains.

Since the SWM device is designed to integrate intimately on the skin, it is important to understand its antenna performance on the skin for achieving reliable and long-range wireless communications when a user wears it. At close proximity, human body tissues can cause scattering and absorption of the electromagnetic waves transmitted and/or received by the antenna due to higher relative permittivity (e.g., $\varepsilon_{r}=$ 38.1, 10.8, and 52.8 at 2.45 GHz for the skin, fat, and muscle, respectively) of the tissues, which may result in poor wireless connectivity [59,60]. To ensure the proper antenna performance of the SWM device on the skin, a T-shaped impedance matching network was incorporated into the middle of the transmission line, which is a conductive trace between the antenna and Bluetooth module to deliver electromagnetic waves. While observing reflection coefficients of the transmission line using a vector network analyzer (TTR506A, Tektronix, Inc., Beaverton, OR, USA) as shown in Figure S4, the network was experimentally tunned such that the resonant frequency formed at the Bluetooth operating frequency range, 2.40–2.48 GHz, as shown in Figure 2c. Figure 2c also showed that the reflection coefficients were changed when the device was in the air, which suggests why the impedance of the transmission line is required to be optimized when the device is on the skin not in the air. After such efforts, the SWM device enabled seamless wireless data transmission up to 10 m while it was laminated and operated on the skin (see Figure S5 for more details). Figure 2d shows the comparison of received strength indicator (RSSI) values measured when the SWM device was operated on the skin and in the air. Before the measurements, the T-matching network was optimized for the SWM device in the air. The result shows the overall RSSI values of the SWM device on the skin are lower than ones of the SWM device in the air, which indicates there is power loss in electromagnetic waves due the effect of human body tissues, but the device offers the acceptable Bluetooth signal range to monitor motion data from the SWM device using a portable device.

**Figure 3:**
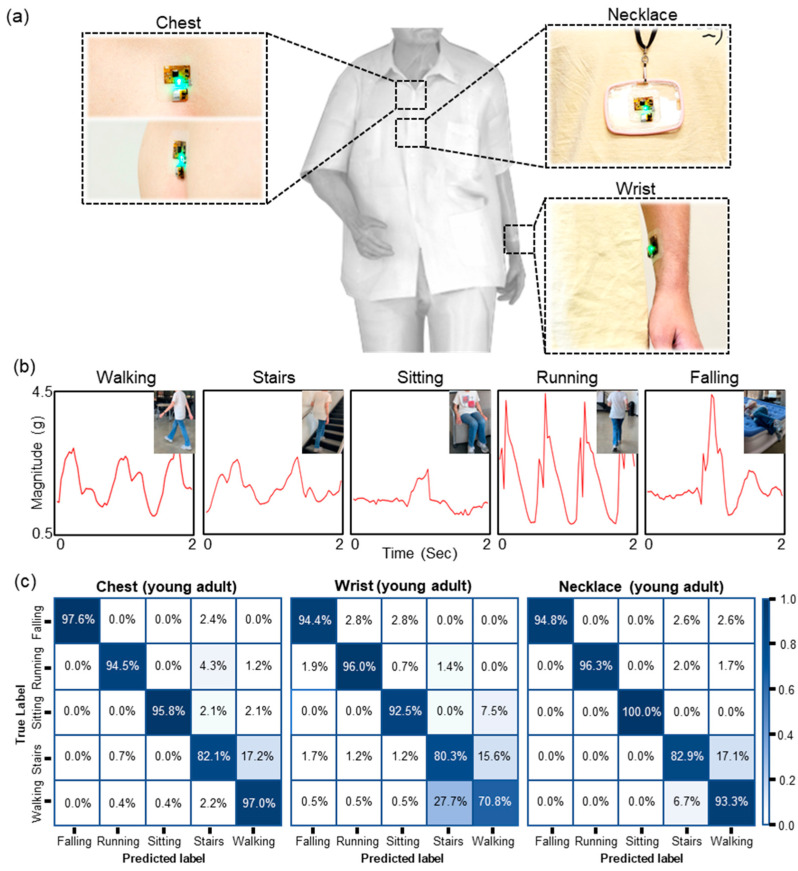
The optimal body location for the device placement. (**a**) Images of the SWM device placed on three different body locations: chest, wrist, and necklace. The device formed intimate integration on the skin without adhesives during the data collection. (**b**) Representative motion data in the form of the magnitude of the acceleration for each activity. (**c**) Confusion matrices obtained from the purposed LSTM model trained and tested using motion data collected from the chest, wrist, and necklace.

**Optimal device location:** One of hypotheses to test in this study is that the accuracy of a DL model to classify human activities can vary based on the SWM device’s locations on the body. During human activities, different body sites produce different motion data [61], so it may be possible that its accuracy trained using motion data collected from a body site is higher than one trained using motion data from other body sites. Therefore, it is necessary to understand the influence of the device’s locations to achieve accurate and reliable fall detection and classification of other activities. For motion data collection to test the hypothesis, three different body sites (chest, wrist, and a form of a necklace) were chosen to place the SWM device on three different young adult groups, respectively, and participants performed five different activities (walking, stairs, sitting, running, and falling). The reason for choosing those locations to place the device is they are the most popular placements for existing wearable-based motion sensors to detect human activities [35,62,63]. Figure 3a includes representative pictures of the device placed on different body sites, and Figure 3b shows representative motion data for each activity based on the magnitude of the acceleration. Due to the high work of adhesion and thin thickness of the device, the device formed intimate integration with the skin during the data collection.

After the completion of the data collection, three different LSTM models were trained and tested independently using XYZ of acceleration and gyroscope features of the chest, wrist, and necklace motion data, and Figure 3c shows their results in a confusion matrix. The accuracy of approx. 97.6%, 94.4%, and 94.8% for the fall detection was achieved, and the overall accuracy of approx. 93.2%, 82.4%, and 92.2% for human activity classification was achieved using motion data from the chest, wrist, and necklace, respectively. In addition to accuracy matrices presented in Figure 3c, average accuracy, precision, recall, and F1-score of those models trained and tested on the chest, wrist, and necklace dataset collected from young adults are shown in Table 2. It must be noted that the optimization of DL models along with different input datasets are discussed in detail later, and results in Figure 3c were present after finalizing the DL model with the input dataset. From the results, it can be observed that the LSTM model trained using motion data collected from the chest performs better than others in predicting all kinds of activities including fall. Further investigations are required, but one of possible reasons with lower accuracies of those models may be due to additional movements generated from the locations, which can serve as noises. According to the visual observations during the motion data collection, each body location where the device was placed demonstrated different movements. For example, most participants swung their arms in slightly different ways, while the whole body movement showed a sinusoidal pattern in the vertical and lateral directions as it moved forward [64]. Even though the necklace was placed near the chest, the necklace shook left and right while walking, and it often flied away from the body when the physical activity level of participants increased. Overall, it is a reasonable inference that motion data from the wrist and necklace can be more complex compared with one from the chest.

**The optimization of DL models:** Five different types of DL models (LSTM, CNN-1D, ConvLSTM-1D, Bi-LSTM, and CNN-LSTM) and four different types of motion input datasets (Mag. of acc., XYZ acc., XYZ acc. and gyro., and combined) were investigated to understand an optimal DL model and input dataset with the SWM device for fall and other human activity classifications. Those five DL models are commonly used in other studies related to human activity classification including fall detection [65,66], and the classification accuracy of models can vary with types of input datasets. If a DL model trained using an input dataset with less attributes performs better, it can lead to reducing the device cost and computational time to train the DL model with a large-scale dataset to enhance the classification accuracy. In this experimental study, the motion data collected from the young adults’ chest was utilized to train the DL models. A grid search over a wide range of hyperparameters with 10-fold cross-validation was performed to find the best-performing architecture of the DL models (see Table S3 for more details). The overall accuracies obtained from four different input datasets on all five human activities with five different DL models are shown in Table 3. The highest value in each column is shown in bold. From the results, all DL models show its highest accuracy of human activity classification is more than 90%, but the LSTM model trained on the XYZ acc. and gyro. dataset shows better performance than combinations of other DL models and input datasets. The LSTM with XYZ acc. input dataset showed (more than 92% accuracy) the second highest accuracy, which suggests a simple and cost-effective motion sensor (e.g., a XYZ accelerometer) can be sufficient to achieve an acceptable accuracy of human activity classification. For this study, however, we used the LSTM model with XYZ acc. and gyro. input dataset since the combination provided the highest accuracy. The selected architecture and hyperparameters of other models used are presented in Table S1.

**Figure 4:**
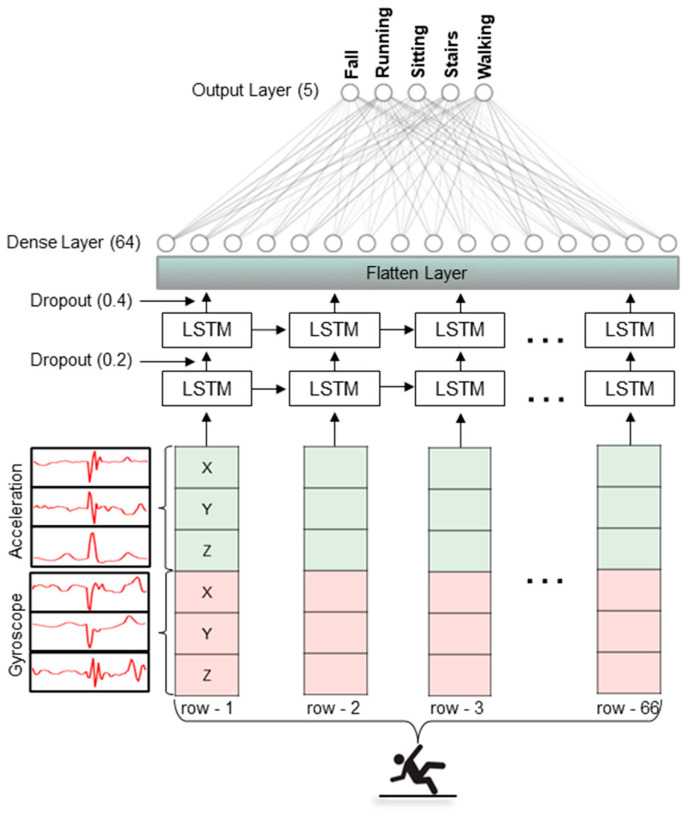
Overall architecture of the LSTM-based classification model used this study. The input was fed to two LSTM layers followed by dropouts for regularization purposes. Finally, outputs from all the LSTM units were merged together in a one-dimensional vector using a flatten layer which was followed by a hidden layer with 64 neurons.

The details of the input layer, intermediate layers, and output layers for the optimized LSTM model are illustrated in Figure 4. Briefly, the LSTM model contained two LSTM layers with 256 memory units and a fully connected layer. For regularization purposes, dropouts of 20% and 40% were used to reduce the complexity of the model and prevent overfitting. It must be noted that the input to the model is 66 sequential records (2 frames with 33 sequences in a frame) and has six variables (X, Y, and Z coordinates of accelerometer and gyroscope). The input was fed to two LSTM layers followed by dropouts. Finally, outputs from all the LSTM units were merged together in a one-dimensional vector using a flatten layer which was followed by a hidden layer with 64 neurons. Finally, an output layer with five neurons was used and activated by the softmax function.

**Figure 5:**
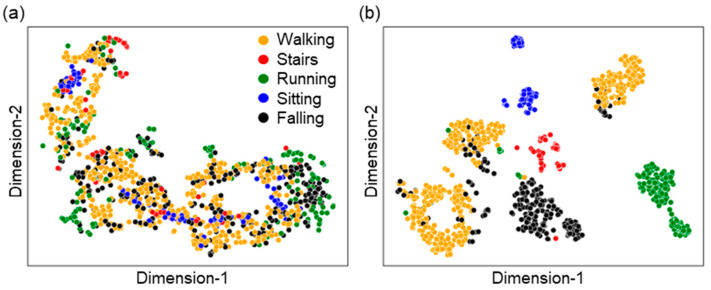
t-SNE to visualize the high-dimensional embedding learned by different features. (**a**) Raw features: XYZ acc. And gyro., 6 attributes (learning rate: 20, perplexity: 40) (**b**) after training the LSTM model, features taken from last hidden layer (learning rate: 10, perplexity: 30).

**The t-SNE visualizations of features before and after training:** t-Distributed Stochastic Neighbor Embedding (t-SNE) is an unsupervised, non-linear transformation algorithm commonly used for exploring high-dimensional data. To observe the effectiveness of the model we proposed, we visualized a two-dimensional feature space obtained from the t-SNE algorithm [67] for raw features and for the features obtained from an intermediate layer of the final model after training. The two-dimensional visualization obtained from applying tSNE to the features obtained from the second last layer of the LSTM model after training on adult chest data can be observed. Since the t-SNE algorithm is highly sensitive to the hyperparameters (learning rate and perplexity), both of the plots shown in Figure 5 were optimized by observing a wide range of possible combinations. From Figure 5, it can be observed that class separation is remarkably improved after training the model. It is not always the case that class separation can be visualized in two-dimensional space, but it provides a basic idea of how our selected feature set and model perform.

**Figure 6:**
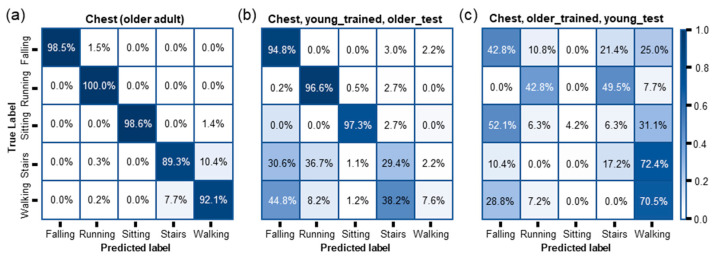
Confusion matrices obtained from the purposed LSTM model. (**a**) Trained on older adult chest data, tested on older adult chest data; (**b**) trained on young chest data and tested on older adult chest data; (**c**) trained on older adult chest data, tested on young chest data.

**Cross-testing of the DL models trained using motion data from different age groups**: After finalizing the best-performing model architecture and input dataset from cross-validation results, three different LSTM models were trained independently using chest, wrist, and necklace motion data from young adults. The results of the models are already discussed above in Figure 3c. Furthermore, a similar LSTM model was trained and tested using the chest motion data collected from older adults (see Table 1 for more details). Figure 6a shows the results in a confusion matrix. The accuracy of approx. 98.5% for falls was achieved, and the overall accuracy of approx. 94.4% for all human activity classification was achieved. Compared with the results based on the chest motion data from young adults (the accuracy of approx. 97.6% for fall and the overall accuracy of approx. 93.3% for all human activities), classifying fall and other human activities for older adults using the SWM device looks very promising. Another hypothesis to test in this study is that a DL model trained using motion data collected from young adults is not compatible to classify motion data collected from older adults because of the differences in human movements between young and older adults due to the body changes with aging. For this experimental study, the test datasets were switched and fed to different age group’s LSTM models. For example, the test dataset from young adults’ chest was fed to the LSTM model trained using older adult chest data. Figure 6b,c show the results in a confusion matrix. The accuracy for fall and overall accuracy for all human activity classification decreased to approx. 94.8% and approx. 48.3%, respectively, when the model is trained on young adult chest data and tested against older adult chest data. Similarly, the accuracy for fall and overall accuracy for all human activity classification decreased to approx. 42.8% and approx. 46.97%, respectively, when the model trained on older adult chest data tested against young adult chest data. While the accuracy of fall detection of the model trained on young adult chest data and tested against older adult chest data was acceptable, it is observed that both models failed to achieve higher accuracy viz. a model trained on young adult chest data and tested against older adult chest data as well as a model trained on older adult chest data and tested on young adult chest data. In addition to the individual class accuracies shown in Figure 6, the other performance metrices including average accuracy, average precision, average recall, and average F1-score are presented in Table 4. Another hypothesis to test in this study is that a DL model trained using motion data collected from young adults is not compatible to classify motion data collected from older adults because of the differences in human movements between young and older adults due to the body changes with aging.

**User Perceptions of the SWM device:** To understand the wearability of the SWM device, the survey of user perceptions was conducted after each participant in the older adult group completed the motion data collection using the device. The survey targeted only the older adult population since the long-term goal of the study is to develop a comfortable and reliable fall detection system using the SWM device for older adults, so it is important to understand their perceptions on the device. The survey was administered by using face-to-face interviews, and each survey lasted about 20 min. The survey date was transferred to IBM SPSS Statistics 28 for analysis. The survey included five questions from the questionnaire measuring user acceptance of wearable devices to see whether the SWM device was comfortable, convenient, important, useful, and safe to participants [68]. Responses to each item were scored using a 4-point Likert-type scale, ranging from “strongly disagree” to “strongly agree,” with higher averaged scores indicating a greater endorsement of the construct as shown in Table 5. The mean score of use perceptions of the device was 23.6 (SD = 0.55; Range = 23 to 24). This indicates that participants have highly positive perceptions of using the SWM device. However, a participant’s comment indicated that the device should have better adherence. This may be due to excessive sweating during activities, which weakens the adhesion strength of the device and causes the device fall off from the skin. Regarding participants’ socio-demographic characteristics, the age of participants was ranged from 62 to 69 years, with a mean age of 65.4 years. There were more females (60%) than males (40%). A total of 80% of participants were married and had received a bachelor’s degree or higher. The majority of participants perceived that they had good (40%) or very good (60%) health conditions.

## 4. Conclusions

In the present work, a comfortable, cost-effective, and reliable fall detection system for older adults is successfully introduced. Due to its ultrathin and flexible design, our skin-wearable motion monitoring device exhibited an intimate integration to the skin without any adhesives. It demonstrated good flexibility, and the skin-wearability of the device was confirmed through the user perception survey taken by older adults who participated in this study. The antenna design of the device was also carefully optimized to provide seamless wireless communication even when participants wore the device on the skin and performed various human activities. Furthermore, to develop an optimal DL model to classify falls and other human activities, various DL models, input datasets, and body locations for the device placement were explored. While the performances of the combination of some models and input datasets were comparable, the LSTM model trained on the XYZ acc. and gyro. input dataset demonstrated the highest accuracy of 97.6% and 98.5% in fall detection for young adults and older adults, respectively. The chest was found as the optimal location to place the SWM device. In addition, the results from the cross-testing of the DL models trained using motion data from different age groups suggested large motion databases directly collected from older adults may be essential to improve the sensitivity and specificity of DL models for reliable fall and other human activity classifications for the population of older adults. However, it is essential to encourage active participation of older adults to build such large motion databases. This can be implemented by providing fall risk education, showing why falls are serious public health issues, and how wearable technologies can help to minimize the adverse consequences of falls for older adults. In future works, the effect of physical conditions (e.g., height and weight) of older adults on results of deep learning models will be investigated. The improvement of the breathability of the SWM device will be also explored to ensure better user comfort and device adherence on the skin.

## Figures and Tables

**Table 1:** Overall description of the dataset collected from different body locations of young and older adults where the SWM device was laminated. Each cell includes train/test/total data points in order.

	Device Placement	Walking	Stairs	Running	Sitting	Falling
**Young Adults**	**Chest**	1111	538	363	191	112
		278	134	91	48	28
		1389	672	454	239	140
	**Wrist**	878	693	611	162	139
		220	173	153	40	35
		1098	866	764	202	174
	**Necklace**	1197	746	1193	171	154
		299	187	298	43	38
		1496	933	1491	214	192
**Older Adults**	**Chest**	1015	710	838	148	136
		254	177	210	37	34
		1269	887	1048	185	170

**Table 2:** Comparison of average accuracy, average precision, average recall, and average F1-score of models trained on chest, wrist, and necklace data and tested using chest, wrist, and necklace dataset collected from young adults.

Device Placement	Accuracy	Precision	Recall	F1-Score
**Chest**	0.9326	0.9757	0.9606	0.9674
**Wrist**	0.8244	0.8446	0.8682	0.8537
**Necklace**	0.9225	0.9450	0.9331	0.9387

**Table 3:** The average 10-fold cross-validation accuracy of LSTM, CNN-1D, CNN-LSTM, Conv-LSTM-1D, and Bi-LSTM on four different input datasets from the young adult chest data on five human activities.

Model	Mag. of acc. (*n* = 1)	XYZ acc. (*n* = 3)	XYZ acc. and Gyro. (*n* = 6)	Combined (*n* = 7)
**LSTM**	0.905 ± 0.014	0.928 ± 0.015	0.936 ± 0.011	0.931 ± 0.011
**CNN**	0.915 ± 0.010	0.879 ± 0.015	0.931 ± 0.009	0.927 ± 0.008
**CNN-LSTM**	0.903 ± 0.026	0.921 ± 0.022	0.925 ± 0.022	0.921 ± 0.018
**Conv-LSTM**	0.887 ± 0.021	0.926 ± 0.013	0.885 ± 0.046	0.866 ± 0.038
**Bi-LSTM**	0.886 ± 0.019	0.914 ± 0.030	0.912 ± 0.017	0.909 ± 0.023

**Table 4:** Comparison of average accuracy, average precision, average recall, and average F1-score of models trained on older adult chest data and tested on older adult chest data, trained on young chest data and tested on older adult chest data, and trained on older adult chest data and tested on young chest data, respectively.

Device Placement	Training Dataset	Test Dataset	Accuracy	Precision	Recall	F1-Score
** Chest **	Old	Old	0.9353	0.9552	0.9578	0.9561
	Young	Older	0.4831	0.5789	0.6565	0.4922
	Older	Young	0.4697	0.5657	0.3550	0.3335

**Table 5:** Responses of the survey of user perception from participants (*n* = 5).

Question	Strongly Disagree	Disagree	Agree	Strongly Agree
**Do you think that the SWM device is comfortable?**	0%	0%	20%	80%
**Do you think that the SWM device is convenient?**	0%	0%	26.7%	73.33%
**Do you think that the SWM device is important?**	0%	6.67%	13.33%	80%
**Do you think that the SWM device is safe?**	0%	0%	20%	80%
**Do you think that the SWM device is useful?**	0%	6.67%	20%	73.33%

## Data Availability

The data and other resources used in this study will be publicly available at https://github.com/KCLabMTU/fall-monitoring.

## Supplementary Materials

The following supporting information can be downloaded at: https://www.mdpi.com/article/10.3390/s23083983/s1, Figure S1: Images of device fabrication process using an ultrathin Cu film; Figure S2: Illustration of the flexible circuit for the SWM device and description of its electronic components; Figure S3: Mechanical bending tests of the SWM device using the hinge fabricated by two glass slides; Figure S4: Experimental setup for the measurement of reflection coefficients of the SWM device; Figure S5: Demonstration of the wireless connection of the SWM device; Table S1: Hyperparameters of Deep Learning Models; Table S2: Number of samples and weights associated with each class for cost-sensitive learning based on motion data from young adults; Table S3: 10-fold cross-validation results of different models and input datasets.
