# Supplementary material for: Experimental Study: Deep Learning-Based Fall Monitoring among Older Adults with Skin-Wearable Electronics
Source: Sensors (Basel). 2023 Apr 14;23(8):3983. doi: 10.3390/s23083983 (PMC10140987; doi:10.3390/s23083983)
Supplement: Supplementary file 1 [file sensors-23-03983-s001.zip › sensors-2311855-supplementary.pdf]

*Supplementary Materials*

# Experimental Study: Deep Learning-Based Fall Monitoring among Older Adults with Skin-Wearable Electronics

**Yongkuk Lee [1,*], Suresh Pokharel [2], Asra Al Muslim [1], Dukka KC [2], Kyoung Hag Lee [3] and Woon-Hong Yeo [4,5]**

[1] Department of Biomedical Engineering, Wichita State University, Wichita, KS 67260, USA; amalmuslim@shockers.wichita.edu
[2] Department of Computer Science, Michigan Technological University, Houghton, MI 49931, USA; sureshp@mtu.edu (S.P.); dbkc@mtu.edu (D.K.)
[3] School of Social Work, Wichita State University, Wichita, KS 67260, USA; kyoung.lee@wichita.edu
[4] George W. Woodruff School of Mechanical Engineering, Georgia Institute of Technology, Atlanta, GA 30332, USA; whyeo@gatech.edu
[5] IEN Center for Human-Centric Interfaces and Engineering, Georgia Institute of Technology, Atlanta, GA 30332, USA

* Correspondence: yongkuk.lee@wichita.edu; Tel.: +1-316-978-7670

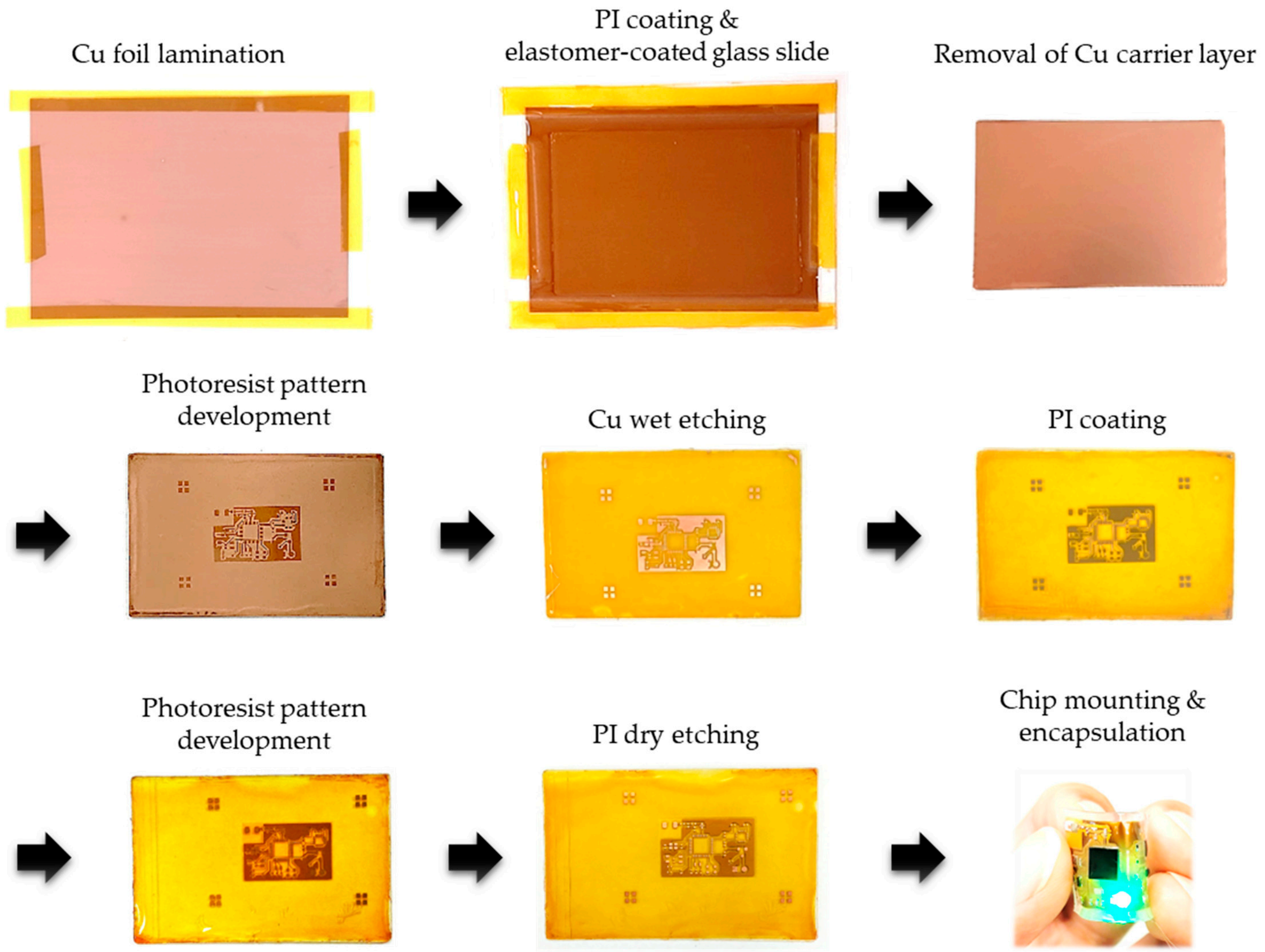


**Figure S1.** Images of device fabrication process using an ultrathin Cu film. The fabrication process includes the lamination of an ultrathin Cu film, photolithography, wet/dry etching, chip mounting, and thin elastomeric encapsulation.

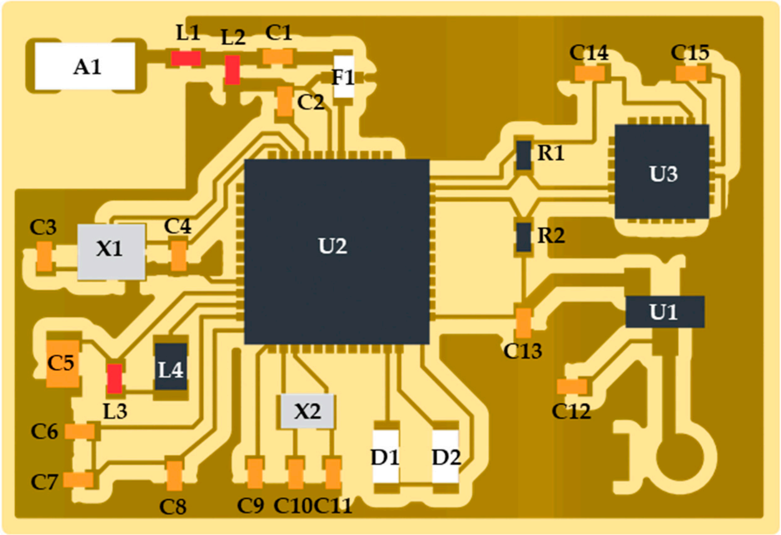


| Annotation | Part description |
|---|---|
| A1 | 2.45 GHz ceramic antenna |
| F1 | Impedance matched low pass filter |
| U1 | 3.3 V voltage regulator |
| U2 | nRF52832 |
| U3 | MPU-9250 |
| X1 | 32 MHz crystal |
| X2 | 32.768 kHz crystal |
| D1 | Green LED |
| D2 | Blue LED |
| L1 | 2.7 nH |
| L2 | 3.9 nH |
| L3 | 15 nH |
| L4 | 10 µH |
| R1, R2 | 10 kΩ |
| C1 | 5 pF |
| C2 | 100 pF |
| C3, C4, C10, C11 | 12 pF |
| C5, C7, C12 | 1 µF |
| C6, C9, C13, C14, C15 | 100 nF |
| C8 | 4.7 µF |

**Figure S2.** Illustration of the flexible circuit for the SWM device and description of its electronic components.

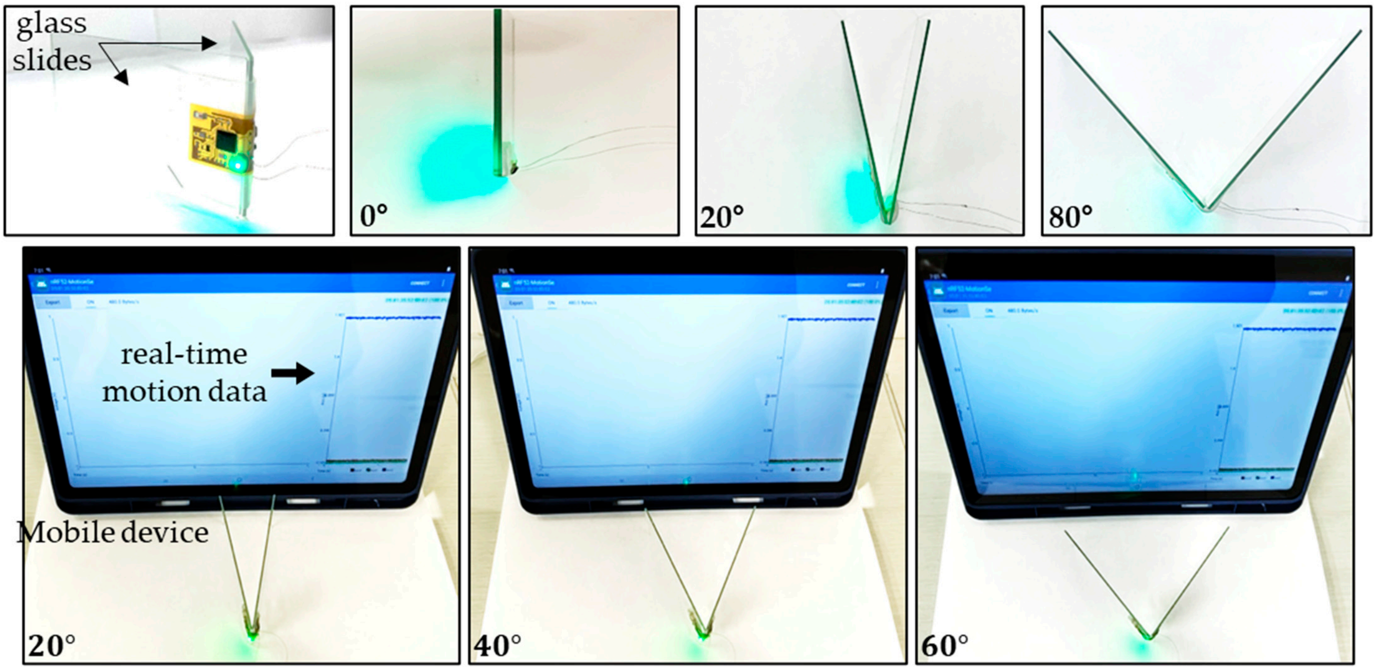


**Figure S3.** Mechanical bending tests of the SWM device using the hinge made by two glass slides. The minimum bending radius of the device was measured as approx. 1 mm. During the bending tests, the device was connected to a Tablet PC for wireless data transmission.

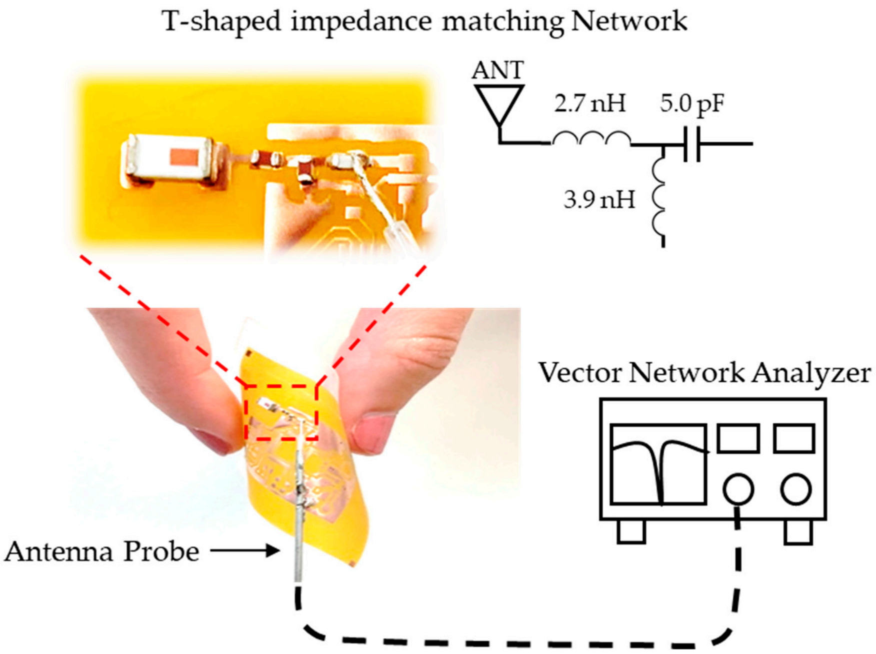


**Figure S4.** Experimental setup for the measurement of reflection coefficients of the SWM device. The T-shaped impedance matching network was incorporated on the middle of the transmission line. The network was experimentally tunned such that the resonant frequency formed at the Bluetooth operating frequency range, 2.40 – 2.48 GHz.

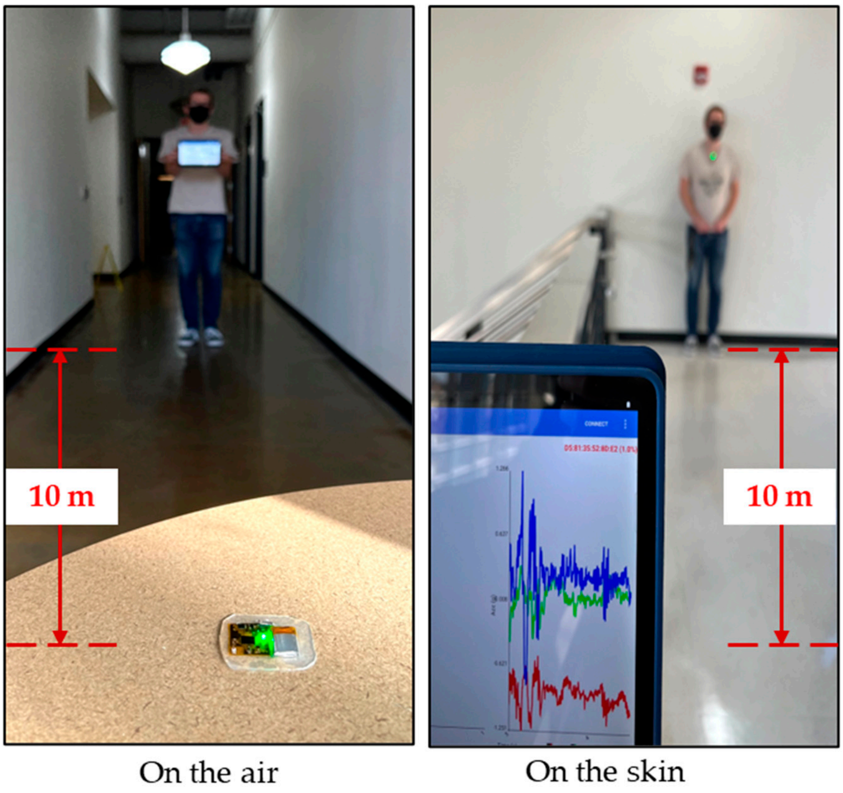


**Figure S5.** Demonstration of the wireless connection of the SWM device. The T-shaped impedance matching network was separately optimized, but the SWM device enabled seamless wireless data transmission up to 10 meters for both when the device was operated on the air and skin.

**Table S1.** Hyperparameters of deep learning Models

| **Name of Model** | **Hyperparameters** |
|---|---|
| LSTM | No. of LSTM layers: 2<br>No. of units in layer 1: 256<br>No. of units in layer 2: 256<br>No of dense layer: 1<br>No. of neurons in hidden layer: 64<br>Optimizer: Adam (Initial learning rate: 0.0001)<br>Loss function: sparse_categorical_crossentropy<br>Intermediate layer's activation function: ReLU<br>Final layer's activation function: Softmax<br>Regularization Method: Dropout<br>No. of trainable parameters: 475,013 |
| CNN-1D | No. of Conv1D layers: 2<br>Conv1D (filters=128, kernel_size=2)<br>MaxPool1D(pool_size=2))<br>Conv1D (filters=128, kernel_size=2)<br>No. of Maxpooling layer: 1<br>MaxPool1D (pool_size=2))<br>No. of LSTM layer: 1<br>LSTM (units=256, input_shape= X_train[0].shape, return_sequences=True)<br>No of dense layer: 1<br>No. of neurons in hidden layer: 128<br>Optimizer: Adam (Initial learning rate: 0.0001)<br>Loss function: sparse_categorical_crossentropy<br>Intermediate layer's activation function: ReLU<br>Final layer's activation function: Softmax<br>Regularization Method: Dropout<br>No. of trainable parameters: 126,837 |
| CNN-LSTM | No. of Conv1D layers: 1<br>Conv1D (filters=256, kernel_size=2)<br>No. of Maxpooling layer: 1<br>MaxPool1D (pool_size=2)<br>No. of LSTM layer: 1<br>LSTM (units=256, input_shape= X_train[0].shape, return_sequences=True)<br>No of dense layer: 1<br>No. of neurons in hidden layer: 128<br>Optimizer: Adam (Initial learning rate: 0.0001)<br>Loss function: sparse_categorical_crossentropy<br>Intermediate layer's activation function: ReLU<br>Final layer's activation function: Softmax<br>Regularization Method: Dropout<br>No. of trainable parameters: 562,181 |

| | |
|---|---|
| ConvLSTM-1D | No. of ConvLSTM layers: 2<br>No. of intermediate dense layer: 1<br>No of Neurons in hidden dense layer 1: 64<br>Optimizer: Adam (Initial learning rate: 0.0001)<br>Loss function: sparse_categorical_crossentropy<br>Intermediate layer's activation function: ReLU<br>Final layer's activation function: Softmax<br>Regularization Method: Dropout<br>No. of trainable parameters: 828,101 |
| Bi-LSTM | No. of Bi-LSTM layers: 2<br>No. of units Bi-LSTM layer 1: 256<br>No. of units Bi-LSTM layer 2: 256<br>No of dense layer: 1<br>No. of neurons in hidden layer: 64<br>Optimizer: Adam (Initial learning rate: 0.0001)<br>Loss function: sparse_categorical_crossentropy<br>Intermediate layer's activation function: ReLU<br>Final layer's activation function: Softmax<br>Regularization Method: Dropout<br>No. of trainable parameters: 2,136,443 |

* Other hypermeters not mentioned above are taken default from Keras API. The number of layers and filter size were adjusted for fewer features (1 and 3). No. of parameters are based on 6 input configurations. The approximate training time for these models varied between 15 to 20 minutes for our dataset on a Dell Precision 7920 Workstation with Intel® Xeon(R) Silver 4216 CPU @ 2.10GHz x 32 processor and Quadro RTX 4000 graphics card.

**Table S2.** Number of samples and weights associated with each class for cost-sensitive learning based on motion data from young adults.

| Class | Walking | Stairs | Running | Sitting | Falling |
|---|---|---|---|---|---|
| **Number of Samples** | 1389 | 672 | 454 | 239 | 140 |
| **Class Weight** | 0.4167 | 0.8613 | 1.2748 | 2.4210 | 4.1342 |

**Table S3.** 10-fold cross-validation results of different models and input datasets.

| Mag. of Acc. (n=1) | | | | | |
|---|---|---|---|---|---|
| **Fold** | CNN-1D | LSTM | BI-LSTM | ConvLSTM-1D | CNN-LSTM |
| **1** | 0.9181034483 | 0.9094827586 | 0.875 | 0.9051724138 | 0.9137931034 |
| **2** | 0.9125862069 | 0.9310344828 | 0.900862069 | 0.8922413793 | 0.9051724138 |
| **3** | 0.9267241379 | 0.8879310345 | 0.875 | 0.9094827586 | 0.8793103448 |
| **4** | 0.9267241379 | 0.9094827586 | 0.9181034483 | 0.8706896552 | 0.8965517241 |
| **5** | 0.9153448276 | 0.9120689655 | 0.849137931 | 0.8577586207 | 0.8577586207 |
| **6** | 0.9264069264 | 0.9047619048 | 0.9004329004 | 0.8961038961 | 0.9220779221 |
| **7** | 0.9004329004 | 0.8831168831 | 0.8744588745 | 0.9177489177 | 0.9437229437 |
| **8** | 0.9004329004 | 0.8961038961 | 0.8787878788 | 0.8614718615 | 0.8744588745 |
| **9** | 0.9123809524 | 0.9134199134 | 0.8917748918 | 0.8787878788 | 0.9220779221 |
| **10** | 0.9107359307 | 0.9004329004 | 0.8917748918 | 0.8787878788 | 0.9177489177 |
| **Mean** | 0.9149872369 | 0.9047835498 | 0.8855332886 | 0.886824526 | 0.9032672787 |
| **SD** | 0.00983218260 | 0.01377863641 | 0.01917859355 | 0.02054507621 | 0.02622229183 |
| **XYZ Acc. (n=3)** | | | | | |
| **Fold** | CNN-1D | LSTM | BILSTM | ConvLSTM-1D | CNN-LSTM |
| **1** | 0.8793103448 | 0.9137931034 | 0.9051724138 | 0.9267241379 | 0.9353448276 |
| **2** | 0.8836206897 | 0.9396551724 | 0.9353448276 | 0.9396551724 | 0.9482758621 |
| **3** | 0.8879310345 | 0.9310344828 | 0.9353448276 | 0.9353448276 | 0.9267241379 |
| **4** | 0.875 | 0.9353448276 | 0.9525862069 | 0.9310344828 | 0.8706896552 |
| **5** | 0.8405172414 | 0.9525862069 | 0.9224137931 | 0.9120396552 | 0.9267241379 |
| **6** | 0.8831168831 | 0.9393939394 | 0.9350649351 | 0.9393939394 | 0.9393939394 |
| **7** | 0.8787878788 | 0.9090909091 | 0.9090909091 | 0.9267241379 | 0.9220779221 |
| **8** | 0.8961038961 | 0.9177489177 | 0.8571428571 | 0.9047619048 | 0.9264069264 |
| **9** | 0.8874458874 | 0.9350649351 | 0.9134199134 | 0.9350649351 | 0.9047619048 |
| **10** | 0.8787878788 | 0.9090909091 | 0.8701298701 | 0.9090909091 | 0.9090909091 |
| **Mean** | 0.8790621735 | 0.9282803403 | 0.9135710554 | 0.9259834102 | 0.9209490222 |
| **SD** | 0.01482868659 | 0.01492371894 | 0.03013389952 | 0.01288244924 | 0.0219104719 |
| **XYZ Acc. & Gyro. (n=6)** | | | | | |
| **Fold** | CNN-1D | LSTM | BILSTM | ConvLSTM-1D | CNN-LSTM |
| **1** | 0.9396551724 | 0.9396551724 | 0.9137931034 | 0.9267241379 | 0.9094827586 |
| **2** | 0.9439655172 | 0.9439655172 | 0.9181034483 | 0.9137931034 | 0.9525862069 |
| **3** | 0.9382758621 | 0.9324137931 | 0.9094827586 | 0.8879310345 | 0.9396551724 |
| **4** | 0.9396551724 | 0.9396551724 | 0.9267241379 | 0.9051724138 | 0.9482758621 |

| | | | | | |
|---|---|---|---|---|---|
| **5** | 0.9224137931 | 0.9396551724 | 0.9094827586 | 0.8448275862 | 0.9051724138 |
| **6** | 0.9264069264 | 0.9437229437 | 0.9220779221 | 0.9177489177 | 0.9393939394 |
| **7** | 0.9177489177 | 0.9480519481 | 0.9220779221 | 0.9047619048 | 0.9090909091 |
| **8** | 0.9307359307 | 0.9177489177 | 0.8701298701 | 0.8917748918 | 0.8831168831 |
| **9** | 0.9223809524 | 0.9177489177 | 0.9264069264 | 0.8874458874 | 0.9264069264 |
| **10** | 0.9264069264 | 0.9393939394 | 0.9047619048 | 0.7705627706 | 0.9350649351 |
| **Mean** | 0.9307645171 | **0.936201149**4 | 0.9123040752 | 0.8850742648 | 0.9248246007 |
| **SD** | 0.00904419271 | 0.01053355872 | 0.01661726774 | 0.04620006843 | 0.0222833058 |
| **Combined features (n=7)** | | | | | |
| **Fold** | CNN-1D | LSTM | BILSTM | ConvLSTM-1D | CNN-LSTM |
| **1** | 0.9224137931 | 0.9482758621 | 0.9310344828 | 0.8793103448 | 0.9267241379 |
| **2** | 0.9310344828 | 0.9396551724 | 0.9224137931 | 0.8405172414 | 0.9439655172 |
| **3** | 0.9353448276 | 0.9267241379 | 0.9137931034 | 0.8879310345 | 0.9353448276 |
| **4** | 0.9439655172 | 0.9396551724 | 0.875 | 0.9439655172 | 0.9267241379 |
| **5** | 0.9181034483 | 0.9224137931 | 0.9224137931 | 0.8318965517 | 0.9224137931 |
| **6** | 0.9264069264 | 0.9264069264 | 0.9090909091 | 0.8701298701 | 0.9437229437 |
| **7** | 0.9177489177 | 0.9393939394 | 0.9264069264 | 0.8268398268 | 0.9090909091 |
| **8** | 0.9220779221 | 0.9104329004 | 0.8614718615 | 0.8961038961 | 0.9004329004 |
| **9** | 0.9264069264 | 0.9350649351 | 0.9264069264 | 0.8605172496 | 0.8961038961 |
| **10** | 0.9264069264 | 0.9264069264 | 0.9004329004 | 0.8225108225 | 0.9047619048 |
| **Mean** | 0.9269909688 | 0.9314429766 | 0.9088464696 | 0.8659722355 | 0.9209284968 |
| **SD** | 0.00806079446 | 0.01100758952 | 0.02348154318 | 0.03789950829 | 0.01752878326 |
